# Quantification of ferroptosis pathway status revealed heterogeneity in breast cancer patients with distinct immune microenvironment

**DOI:** 10.3389/fonc.2022.956999

**Published:** 2022-09-02

**Authors:** Yuying Li, Tianfu Li, Duanyang Zhai, Chuanbo Xie, Xiaying Kuang, Ying Lin, Nan Shao

**Affiliations:** ^1^ Breast Disease Center, The First Affiliated Hospital, Sun Yat-sen University, Guangzhou, China; ^2^ Laboratory of Surgery, The First Affiliated Hospital, Sun Yat-sen University, Guangzhou, China; ^3^ Cancer Prevention Center, Sun Yat-sen University Cancer Center, State Key Laboratory of Oncology in South China, Collaborative Innovation Center for Cancer Medicine, Guangzhou, China

**Keywords:** programmed cell death, ferroptosis, tumor microenvironment, heterogeneity, NDUFA13

## Abstract

Clinical significance and biological functions of the ferroptosis pathway were addressed in all aspect of cancer regarding multi-omics level; however, the overall status of ferroptosis pathway alteration was hard to evaluate. The aim of this study is to comprehensively analyze the putative biological, pathological, and clinical functions of the ferroptosis pathway in breast cancer on a pathway level. By adopting the bioinformatic algorithm “pathifier”, we quantified five programmed cell death (PCD) pathways (KO04210 Apoptosis; KO04216 Ferroptosis; KO04217 Necroptosis; GO:0070269 Pyroptosis; GO:0048102 Autophagic cell death) in breast cancer patients, and we featured the clinical characteristics and prognostic value of each pathway in breast cancer and found significantly activated PCD in cancer patients, among which ferroptosis demonstrated a significant correlation with the prognosis of breast cancer. Correlation analysis between PCD pathways identified intra-tumor heterogeneity of breast cancer. Therefore, clustering of patients based on the status of PCD pathways was done. Comparisons between subgroups highlighted specifically activated ferroptosis in cluster 2 patients, which showed the distinct status of tumor immunity and microenvironment from other clusters, indicating putative correlations with ferroptosis. NDUFA13 was identified and selected as a putative biomarker for cluster 2 patients. Experimental validations were executed on cellular level and NDUFA13 showed an important role in regulating ferroptosis activation and can work as a biomarker for ferroptosis pathway status. In conclusion, the status of the ferroptosis pathway significantly correlated with the clinical outcomes and intra-tumor heterogeneity of breast cancer, and NDUFA13 expression was identified as a positive biomarker for ferroptosis pathway activation in breast cancer patients.

## Introduction

Programmed cell death (PCD) has long-demonstrated significance in all aspect of life and keeps growing with the discovery of new functions and identification of new classifications (1). The newest subroutines were identified based on the molecular characteristics including apoptosis, necroptosis, pyroptosis, ferroptosis, entotic cell death, netotic cell death, parthanatos, lysosome-dependent cell death, autophagy-dependent cell death, alkaliptosis, and oxeiptosis, of which the molecular function and regulatory mechanism were summarized in a review done by Tang et al. (2).

Numerous reports were seen regarding the pathological alteration of PCD pathways in cancers. Several core genes were identified in the regulatory network of cell death. The caspases family plays a critical role in the regulation of cell death. PCD can be further categorized into two groups: caspase-dependent (e.g., apoptosis and pyroptosis) and caspase-independent (e.g., necroptosis, ferroptosis, parthanatos, alkaliptosis, and oxeiptosis) (3, 4). Studies focusing on the regulatory genes of PCD gave rise to the importance of each pathway in cancer; however, these studies can hardly reflect the overall status alteration of pathways. Quantifications of each pathways in patients were also rarely discussed.

The recent development of algorithms using sequencing data can transform gene-level data into pathway-level data. With considerations of regulatory information between genes, it can accurately reflect the status alteration of each pathway under different conditions and provide novel perspectives in the integration of multi-omics data on cancer (5, 6). Compared to R package “ssGSEA”, which was developed using the annotated gene list of pathways, “pathifier” demonstrated better accuracy in a context-specific manner, especially with novel pathways or self-defined gene lists (7, 8), and was widely used in cancer studies focusing on pathway-based evaluations of pathological effects and identifications of testable biomarkers (6, 9, 10).

To fully understand the characteristics of ferroptosis pathway in breast cancer, we calculated a pathway deregulation score (PDS) of five PCD pathways by adopting “pathifier”, a bioinformatic algorithm, using The Cancer Genome Atlas (TCGA) data. The aim of this study is to comprehensively analyze the putative biological, pathological, and clinical correlations of the ferroptosis pathway in breast cancer on a quantitative pathway level.

## Materials and methods

### Data accession

Expression data from TCGA breast invasive carcinoma (BRCA) dataset by RNA sequencing (RNAseq) (ployA+ IlluminaHiSeq, version 2017-10-13) and relative clinical phenotype information from TCGA Pan-Cancer (PANCAN) Clinical Data Resource (version 2019-12-06) were obtained from UCSC Xena data hub (https://xenabrowser.net/hub/). The expression value was shown as gene-level transcription estimates mean-normalized (per gene) across all TCGA cohorts [PANCAN normalized log2(norm_count+1)]. Four major clinical outcome endpoints were used as pre-described, namely, overall survival (OS), progression-free interval (PFI), disease-free interval (DFI), and disease-specific survival (DSS) (11). GTEx and TCGA PANCAN were also obtained from UCSC Xena data hub. Data from the Molecular Taxonomy of Breast Cancer International Consortium (METABRIC) project and the MSK-IMPACT project were downloaded from the cBioPortal for Cancer Genomics (http://www.cbioportal.org/) and were used as independent external validation datasets (12–14).

### Generation of pathway deregulation score

To evaluate the biological status of PCD pathways in breast cancer, R package “pathifier” was used to transform gene-level information into pathway-level information on the basis of expression data, generating a compact and biologically relevant PDS of each sample (8). PDS ranged from 0 to 1. Tumor samples that got a higher score than the normal sample were deemed highly deregulated, whereas tumor samples with the lower score indicated an inactive status of a certain pathway. A gene list of PCD pathways was extracted from the Kyoto Encyclopedia of Genes and Genomes (KEGG) and Gene Ontology (GO) database (KO04210 Apoptosis; KO04216 Ferroptosis; KO04217 Necroptosis; GO:0070269 Pyroptosis; GO:0048102 Autophagic cell death). Each pathway was running for 1000 attempts with the minimal allowed standard deviation of 0.4. Illustration of clinical relevance was done with “pheatmap”.

### Evaluation of clinical significance

Prognostic value regarding the four clinical outcome endpoints enrolled was done using univariate and multivariate Cox regression. For multivariate Cox regression, PDS was enrolled and forward stepwise regression was done with the ER/PR/HER2 status, age, and T/N/M stage treated as confounders. R package “survival” was used for survival analyses of PDS with best separation cutoffs selected using “survminer”. The minimal proportion of each group was no less than 10%. Further multivariance survival analyses and subgroup analyses were done as described and illustrated using “forestplot”. Hazard ratio (HR) and 95% confidence interval (95%CI) were shown in the forest plot. The Kaplan–Meier plotter (KMplot; http://www.kmplot.com/analysis) was used to evaluate the prognostic value of NDUFA13 in integrative breast patients on both RNA and protein levels. Distance metastasis-free survival was used as an endpoint.

To evaluate the predictive efficiency of PDS, R package “timeROC” was used to construct time-dependent ROC comparing survival data. Time points used include 0, 90, 180, 270, 365, 730, 1,095, 1,825, 2,920, 3,650, and 5,475 days. At each time points included, an AUC value was generated and compared across PCD pathways.

Decision curve analysis (DCA) was done to evaluate whether using the PDS of PCD pathways as prognostic factors would improve clinical decision-making for all decisions (15, 16). The “stdca.R” function was used to conduct DCA with prediction models compared to two default strategies: (1) assume that all patients are tested positive and therefore treat everyone (treat-all) or (2) assume that all patients are tested negative and offer treatment to no one (treat-none). Curves above the treat-none line and outdo treat-all line were deemed to gain benefit. The further assessment focused on the usefulness of the marker to identify patients with and without unnecessary treatment, and the net reduction plot was used to show the intervention avoided based on the prognostic value of the marker.

### Clustering and principle components analysis (PCA)

Correlations between the PDS of PCD pathways were calculated and illustrated using R package “corrgram”. Each pathway was distributed diagonally, with the correlation scatter diagram in the lower left corner and the correlation coefficients in the upper right corner. Further illustrations with circos plot were done using “circlize” (17). All analyses were done using Spearman’s correlation tests.

Clustering of PDS of each PCD pathway was done with patients firstly being divided into “UP”, “DOWN”, and “NO CHANGE” groups using k.means, then clustered with ward.D. Illustrations were done using “pheatmap” and “ggplot2”. PCA was done using “ggord” and illustrated with “yyplot”.

### Function enrichment

Clinical characteristics were compared between clusters using “pheatmap”. Differential expression analyses were done between groups to identify genes that significantly deregulated between clusters using limma (18, 19). For gene set enrichment analysis (GSEA), we predefined the gene rank by expressional correlations. The h.all.v7.4.symbols.gmt and c2.cp.kegg.v7.4.symbols.gmt subsets were used to evaluate the relevant pathways and molecular mechanisms. Based on the predetermined gene rank, the minimum gene set was set to 5, the maximum gene was set to 5000, and 1,000 times of re-sampling. *P*-value < 0.05 and FDR < 0.25 were considered statistically significant.

### Characterizing of tumor microenvironment

To fully present the correlation between PCD pathways and anti-cancer immune response process, cancer immunity was analyzed using the online tool TIP (Tracking Tumor Immunophenotype, http://biocc.hrbmu.edu.cn/TIP/), which integrates “ssGSEA” and “CIBERSORT” for tracking, analyzing, and visualizing the status of the seven-step Cancer-Immunity Cycle using RNAseq data (20). Detailed tumor-infiltrating immune cells were further calculated and characterized with clinical information and previously published TCGA immune subtypes.

Immune cell clustering within subgroups was done to feature the relationship among immune cells in a certain population. Spearman’s correlations with *P*-value less than 0.0001 were left for clustering. Both k.means and hclust were used and integrated with recent studies to generate the final clustering results.

### Selection of putative biomarkers

Exploration of putative biomarkers was first done with differentially expressed genes (DEGs). The calculation of DEGs was done using limma. Selection criteria were adjusted *P*-value < 0.05 and |log2FC| > 1. For DEGs meeting the criteria, ROC was done to calculate the AUC, and genes were further ranged by AUC. Top genes were illustrated in heatmap. Further construction of selective panels was done using Lasso regression. Internal tests were done with patients in TCGA-BRCA 1:1 randomly assigned as a training set and a test set. External independent validations were done in the METABRIC and MSK-IMPACT projects.

### Cell lines

Human breast cancer cell lines MDA-MB-231, SK-BR-3, MDA-MB-468, T-47D, ZR-75-1, and MCF-7 were obtained from American Type Culture Collection (ATCC; Manassas, VA, USA) and cultured in Dulbecco’s modified Eagle’s medium (DMEM; Gibco, New York, USA) or Roswell Park Memorial Institute 1640 (RPMI; Gibco, New York, USA), as required, with 10% fetal bovine serum (FBS; Gibco, New York, USA), and 1% penicillin and streptomycin (Gibco, New York, USA). All cells were cultured in the humidified incubator at 37°C with 5% CO_2_.

### Quantitative real-time polymerase chain reaction (qPCR)

MDA-MB-231 and ZR-75-1 cells were treated with DMSO or RSL3 (MedChemExpress, Monmouth Junction, NJ, USA; 10 μM) for 24 h. Total RNA was extracted through the RNA extraction kit (Promega, Beijing, China) and taken into reverse transcription using PrimeScript RT reagent Kit (Takara, Japan) to produce cDNA under the manufacturer’s instructions. qPCR was conducted with SYBR Premix Ex Taq II (Takara) and LightCycler480 system (Roche, Switzerland). GAPDH was utilized as reference. Primers for NDUFA13 were listed as follow: forward primer: 5′- GGCCCATCGACTACAAACGG-3′; reversed primer: reverse primer: 5′- CGCTCACGGTTCCACTTCATT-3′.

### Reactive oxygen species evaluation

The intracellular level of reactive oxygen species (ROS) was quantified by a Reactive Oxygen Species Assay Kit (Beyotime, China). This kit contains 2’, 7’-dichlorofluorescein-diacetate (DCFH-DA), which is easily oxidized to fluorescent dichlorofluorescein (DCF) by intracellular ROS. Cells were seeded in six-well plates, stimulated with simvastatin for 24 h, then washed with PBS and treated with 10 μM DCFH-DA in the dark for 30 min at 37°C. The fluorescence was observed by fluorescence microscopy at 488-nm excitation and 525-nm emission after being washed three times with PBS.

### Statistical analysis

All analyses were performed using RStudio version 1.2.5033 (R Core Team, Vienna, Austria) statistical software. PDS and gene data were presented as means ± SE unless otherwise indicated. Two group comparisons were made using Student’s t-test, whereas multi-groups comparisons were done with one-way ANOVA. Further comparisons of numerous data were done using chi-square test. All analyses were done with missing sample excluded considering the large sample size. Correlation analyses were done using either Pearson’s test or Spearman’s test depending on parametric or non-parametric distribution of the data. Unless otherwise indicated, a two-sided *P*-value < 0.05 was considered significant. *P < 0.05; **P < 0.01; ***P < 0.001; ****P < 0.0001.

## Results

### Clinical characteristics and prognostic significance of PDS in breast cancer

**Figure 1:**
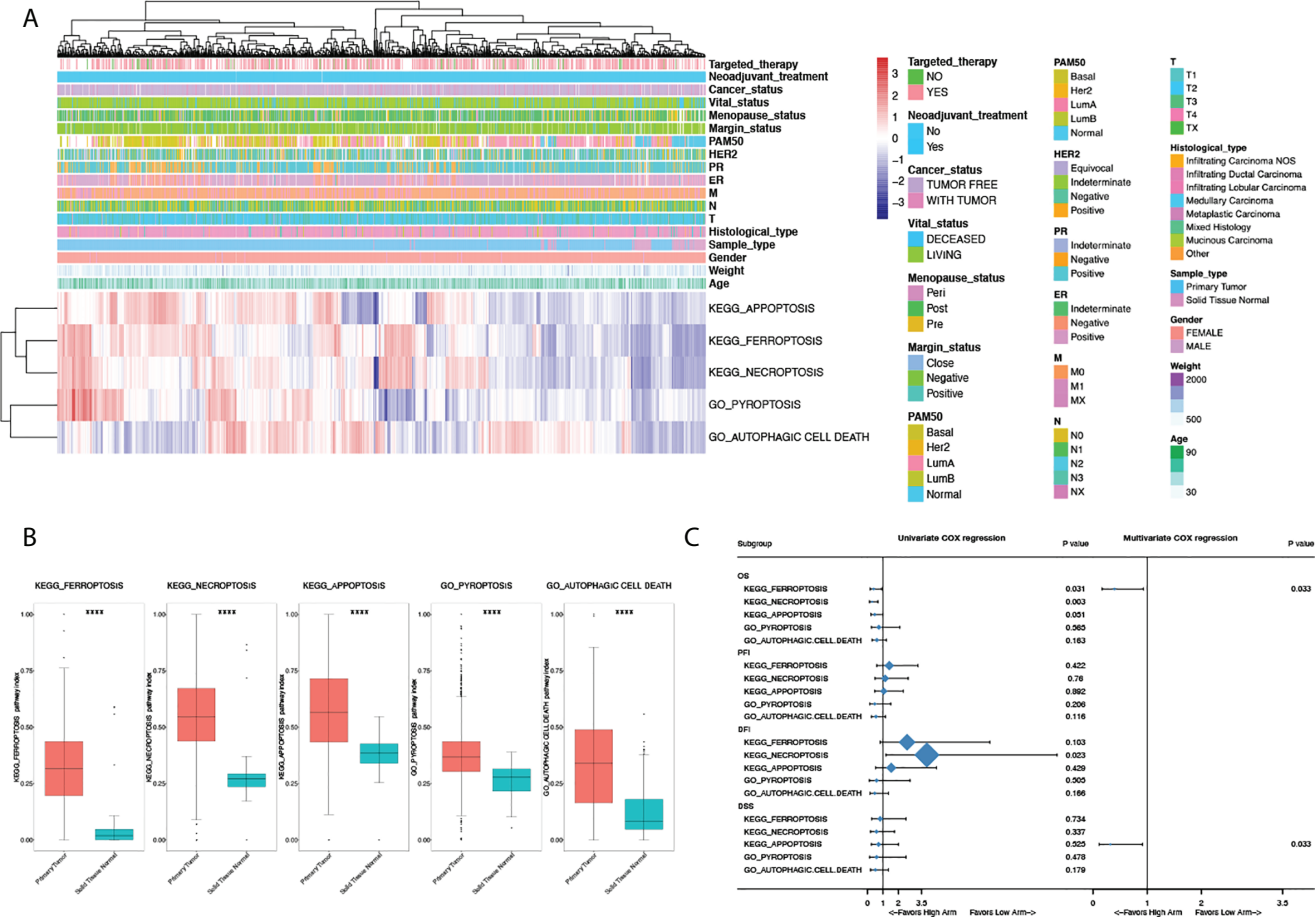
Clinical characteristics and prognostic significance of programmed cell death pathway alterations in breast cancer. **(A)** Heatmap illustrating pathway deregulation score (PDS) of PCD pathways in breast cancer generated with clinical annotations from TCGA BRCA data; colors were row-scaled and zero-centered. **(B)** Comparisons of PDS between sample types. **(C)** Univariate and multivariate Cox regression of PCD pathways in breast cancer regarding overall survival (OS), progression-free interval (PFI), disease-free interval (DFI), and disease-specific survival (DSS). Hazard ratio (HR) and 95% confidence interval (95%CI) were shown in the forest plot. For multivariate Cox regression, all pathway scores were enrolled and forward stepwise regression was done with the ER/PR/HER2 status, age, and T/N/M stage treated as confounders. *P*-value < 0.05 was considered significant. A two-sided *P*-value < 0.05 was considered significant. ****P < 0.0001.

To fully explore the clinical characteristics and prognostic significance of PCD pathway alterations in breast cancer, PDS of PCD pathways (KO04210 Apoptosis; KO04216 Ferroptosis; KO04217 Necroptosis; GO:0070269 Pyroptosis; GO:0048102 Autophagic cell death) for each patient was generated using “pathifier” to quantify the status alteration of each pathway based on RNAseq data from the TCGA-BRCA dataset with 1,091 tumor sample and 113 normal sample (**Figure 1A**). The final status of each PCD pathway was determined by comparing with normal samples (**Figure 1B**). Intriguingly, all PCD pathways were seen hyperactivated in tumor samples (*P* < 0.0001), demonstrating the significant role of PCD in breast cancer. Clinical characterization of PCD pathways was further explored. Among all clinical characteristics included, apoptosis, ferroptosis, and necroptosis pathways were activated in ER, PR-negative, and basal patients, whereas the autophagic cell death pathway was significantly suppressed (**Figure A1A–C**). Interestingly, despite no significant alteration being seen regarding HER2 status, comparisons between PAM50 subtypes showed significantly activated apoptosis, ferroptosis pathways, suppressed pyroptosis, and autophagic cell death pathway in HER2-positive subtype compared to hormone receptor-positive patients (**Figure A1C**). Furthermore, correlations between pathway alterations and vital status revealed significantly activated apoptosis, ferroptosis, necroptosis, and autophagic cell death pathways in living patients compared to deceased patients, demonstrating the putative prognostic significance of PCD in breast cancer (**Figure A1D**).

Given the status of PCD significantly altered between vital status, Cox regression and survival analyses were done to explore the prognostic values of PCD in breast cancer. As shown in **Figure 1C**, ferroptosis and necroptosis are significantly correlated with OS in univariate Cox regression, among which only ferroptosis was identified as an independent prognostic factor in multivariate analysis (HR 0.395, 95%CI 0.198–0.929, P = 0.033). Interestingly, apoptosis significantly correlated with DSS in multivariate analysis with the ER/PR/HER2 status, age, and T/N/M stage was treated as confounders; however, this was not seen in univariate Cox regression.

To validate the prognostic value of PCD, timeROCs were used to compare at a different time point (**Figure A1E**). In accordance with survival analysis, the ferroptosis pathway showed the highest AUC regarding OS, PFI, DFI, and DSS, especially in the first 3 years, with the highest AUC seen in the first year (OS: 0.68; PFI: 0.72; DFI: 0.66; DSS: 0.76) and declining ever after. The prognostic advantage of the ferroptosis pathway persisted in PFI and DSS up to the fifth year. Combined with the clinical significance seen in survival analyses, the ferroptosis pathway has demonstrated putative association with early recurrence and progression of breast cancer, and a certain hypothesis gave rise to the importance of the ferroptosis pathway in breast cancer in both pathogenetic and clinical ways. To further address the putative functions of ferroptosis in clinical decision-making, DCA was done comparing the instructive value of PCD regarding PFI, DFI, and DSS in the first 3 years. Intriguingly, in accordance with timeROC analyses, PDS of the ferroptosis pathway has shown the best efficacy as a risk score for the early recurrence and progression of breast cancer in the first 3 years. In the prediction of PFI, an absolute gaining of Net Benefit (NB) in patients with a threshold probability ranged from 10% to 14%, whereas in DSS, significant gaining of NB was seen in patients with a probability ranging from 6% to 10% (**Figure A1F**). Moreover, evaluation of PDS as therapeutic indicators done in DCA also showed a good efficacy of the ferroptosis pathway regarding PFI and DFI. For patients with a threshold probability ranging from 9.5% to 14%, using PDS of ferroptosis as a biomarker for clinical intervention demonstrated the best efficacy, with a net reduction of 15 per 100 patients. Comparatively, prominent improvement was seen in ferroptosis as a biomarker for DSS in patients with a probability over 6%, with a net reduction of more than a quarter per 100 patients (**Figure A1F**).

### Clustering of breast cancer patients based on PDS

**Figure 2:**
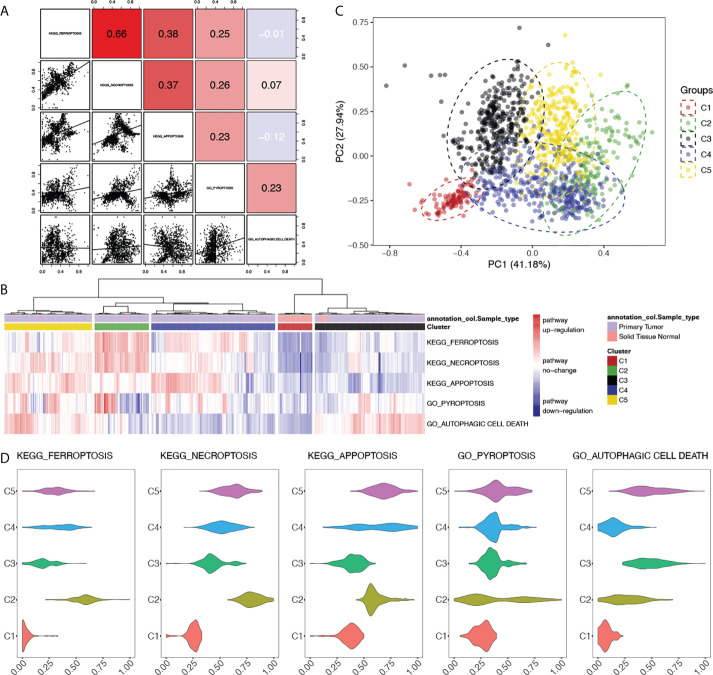
Clustering of breast cancer patients based on the alterations of programmed cell death pathways. **(A)** Correlations between PCD pathways in breast cancer. Correlational R-values between pathways were shown in the upper right with scatter plots in the bottom left. Color was illustrated according to the R-value. Red: R > 0, blue: R < 0. **(B)** Heatmap of clustering based on the PDS of PCD pathways of breast cancer patients with sample type annotation. **(C)** Principle components analysis (PCA) based on the PDS of PCD pathways with clusters of breast cancer patients illustrated with different colors. **(D)** Comparisons of PDS of each PCD pathways between clusters. *P*-value < 0.05 was considered significant.

Numerous works were done focusing on the crosstalk between PCD pathways on both biological and clinical levels; however, studies were done on the gene level, and by generating the PDS of PCD pathways, we tried to quantify the correlation on the pathway level (**Figure 2A**). Positive correlations were found between all PCD, except for autophagic cell death. However, scatter plots of pairwise comparisons between apoptosis, ferroptosis, necroptosis, and pyroptosis revealed underlying heterogeneity among breast cancer samples with the distribution of patients exhibiting distinct clusters. Therefore, clustering based on PDS was done. A total of five clusters were generated as shown in **Figure 2B** with sample types. Normal samples were seen mostly in cluster 1 (Normal = 88, Tumor = 13). PCA also identified a separated distribution between cluster 1 and other clusters (**Figure 2C**). Within tumor samples, PCA has seen a good separation between clusters 2, 3, and 5; however, there is cross-coverage between cluster 4 and other groups. For each pathway, comparisons were made to identify featured alterations in each cluster (**Figure 2D**; **Table A1** **SF4**), among which cluster 2 exhibited significantly hyperactivated ferroptosis and necroptosis pathways and autophagic cell death was significantly activated in cluster 3 and cluster 5. Detailed clinical characteristics were summarized in **Table A2**.

Survival analyses done in each cluster unraveled distinct results between clusters, but good consistency within each cluster (**Figure A1G**). In cluster 2, the activation of ferroptosis and autophagic cell death significantly correlated with better clinical outcomes, whereas cluster 5 has seen the activated PCD indicating worse clinical outcomes. However, in either cluster 2 or cluster 5, identical clinical significance was seen regarding both OS and other outcomes. This suggested that our clustering results successfully eliminate heterogeneity within clusters. Among PCD pathways, ferroptosis showed significant clinical correlations in both cluster 2 and cluster 5 regarding OS, PFI, DFI, and DSS, which supported the clinical significance of ferroptosis in breast cancer addressed above. Furthermore, the different clinical relevance between cluster 2 and cluster 5 draws attention to the putative biological heterogeneity between these two clusters, which needs further exploration.

### Functional enrichment between clusters

To fully explore the biological difference between clusters, clinical characteristics were first summarized and compared in **Table A2**. Clusters consist of different levels of sample type, histological type, ER/PR/HER2 status, M stage, marginal status, vital status, and history of targeted therapy. To explore the different biological characteristics between clusters, biomarkers for each cluster were enriched (**Figure A2A**). Top 100 biomarkers for each cluster were used for functional enrichments. Only specific biomarkers were enrolled for final analysis (**Figure A2B**, **Table A3**). Multi-group enrichments revealed common functions between clusters (**Figures A2C, D**), which mainly focus on the cell cycle pathway. Cluster-specific enrichments showed a distinct biological background of each cluster. Comparatively, cluster 2 specifically enriched in GO:0016579: protein deubiquitination, GO:0061756: leukocyte adhesion to vascular endothelial cell, GO:0046854: phosphatidylinositol phosphorylation, GO:0004842: ubiquitin-protein transferase activity, GO:1901699: cellular response to nitrogen compound, GO:0005667: transcription factor complex, and GO:0019902: phosphatase binding, among which GO:0061756 indicates a putative role in the infiltration of the tumor microenvironment. The differentially enriched pathways provided novel perspectives on the difference between clusters and the intra-tumor heterogeneity, which was mainly discussed in the tumor microenvironment.

**Figure 3:**
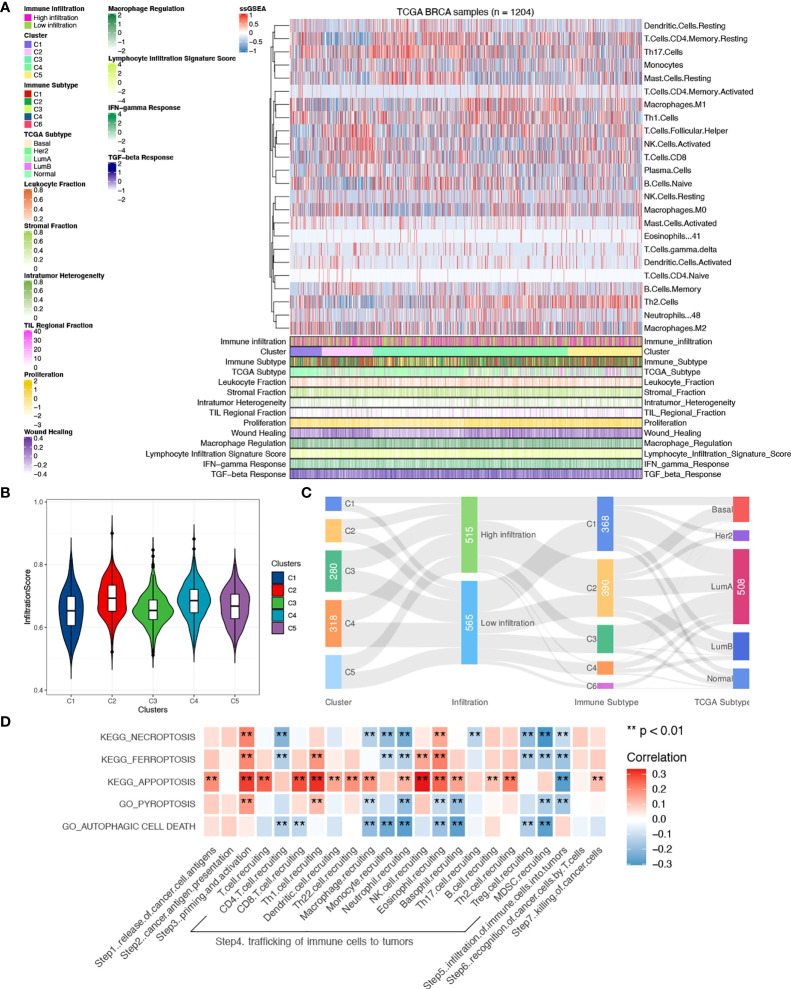
Comparisons of tumor microenvironment between clusters. **(A)** Heatmap of tumor-infiltrating immune cells between clusters with previously categorized immune subtypes information. **(B)** Comparisons of infiltration scores were done between clusters. **(C)** Correlations between clusters and immune subtypes, PAM50, and infiltration levels. **(D)** Correlations of PCD pathways and the seven-step Cancer-Immunity Cycle in all samples. A two-sided *P*-value < 0.05 was considered significant. **P < 0.01.

To explore the underlying correlation mentioned above, characterization of the microenvironment was depicted. A comprehensive heatmap of TILs was shown with patients scored by infiltration level of the 24 immune cells. Previously published immune indexes were also annotated to characterize tumor microenvironment (**Figure 3A**) (21). Leukocyte fraction, stromal fraction, and intra-tumor heterogeneity were used to quantify tumor purity. Comparisons of the overall infiltration score between clusters showed a significantly higher level of infiltration in cluster 2 than other clusters (P < 0.001, **Figure 3B**), which, in accordance with the functional enrichments, demonstrated an hyperactivated immune status in patients with activated ferroptosis pathway. However, compared to previously published immune subtypes and PAM50, cross-links were seen between subgroups and significant consistency was not seen (**Figure 3C**), which proved our clusters a novel classification, and the highlight of cluster 2 indicates a putative regulative role of ferroptosis in tumor immunity.

Given the theory of the seven-step Cancer-Immunity Cycle, clarification of correlations between PCD and specific steps contributes to the understanding of the specific underlying mechanism. Therefore, correlations were done with each step quantified using ssGSEA. As shown in **Figure 3D**, a general relationship was seen between PDC and the seven-step Cancer-Immunity Cycle, among which correlations were mainly found in steps 3, 4, and 5 (**Table A4**). Interestingly, negative correlations were found between pathways and trafficking of immune cells to tumors except for apoptosis, which implies a different biological function between apoptosis and other PCD pathways in immunity.

### Distinct tumor microenvironment between patients with activated and inactivated ferroptosis pathway

**Figure 4:**
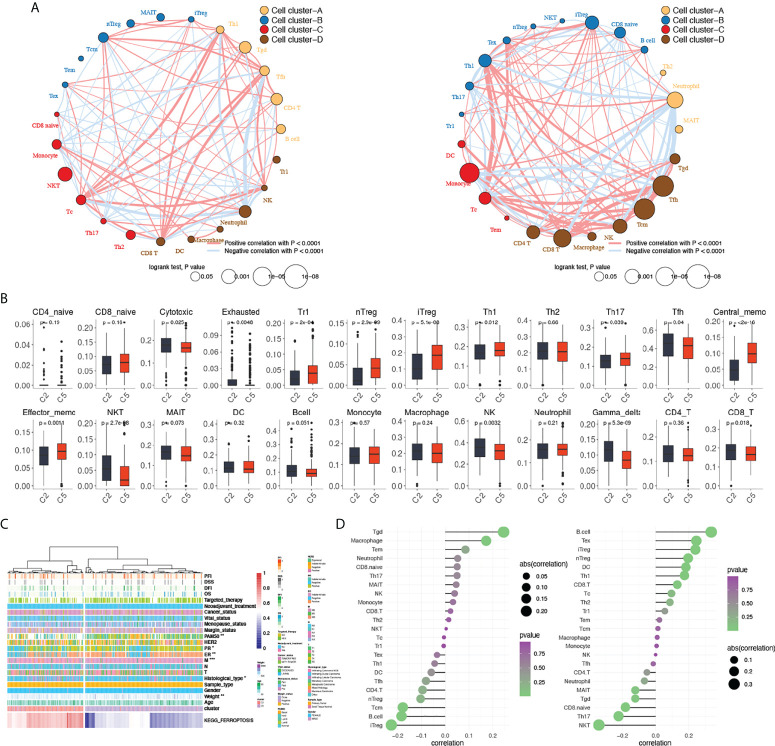
Comparisons of tumor-infiltrating immune cells between cluster 2 and cluster 5. **(A)** Clustering and correlations between tumor-infiltrating immune cells in cluster 2 (left) and cluster 5 (right). Red: positive correlation, blue: negative correlation. **(B)** Comparisons of tumor-infiltrating immune cells between cluster 2 and cluster 5.  **(C)**. Heatmap of KEGG_FERROPTOSIS pathway alteration and clinical characteristics between clusters 2 and 5. **(D)** Correlations between KEGG_FERROPTOSIS and tumor-infiltrating immune cells between cluster 2 (left) and cluster 5 (right). A two-sided *P*-value < 0.05 was considered significant. *P < 0.05; **P < 0.01; ***P < 0.001.

Further exploration of the tumor microenvironment was done focusing on the different intercellular interactions of TILs. Correlations of each cell were calculated and clustered within each group. With the size of each dot representing the survival significance of each cell, cells clustered showed a comprehensive correlation and a different distribution between cluster 2 and cluster 5 (**Figure 4A**). Distinct interactions were seen with TILs clustered differently, demonstrating a distinct tumor microenvironment between clusters 2 and 5 caused by status alteration of ferroptosis. In summary, cluster 2 featured highly infiltrated immune cells that demonstrated anti-tumor efficacy, while cluster 5 exhibited a higher proportion of tumor-promoting cells.

Given the putative regulatory function of PCD mainly focuses on the immune infiltration process, comparisons of 24 tumor-infiltrating immune cells were done between cluster 2 and cluster 5 (**Figure 4B**), in which a lower level of type 1 regulatory T cells (Tr1), natural regulatory T cells (nT_reg_), induced regulatory T cells (iT_reg_), T helper cells 1 (T_H_1), IL-17–producing effector T helper cells (T_H_17), central memory T cell (T_cm_), and effector memory T cell (T_em_) were seen in cluster 2, whereas cytotoxic T cells (Tc), exhausted T cells (T_ex_), natural killer T cells (NKT), follicular helper T cells (Tfh), NK cells, gammadelta T cells (γδT/Tgd), and CD8^+^ T cells (CD8T) are highly infiltrated. The different levels of tumor-infiltrating immune cells indicate a distinct microenvironment between clusters 2 and 5 that may affect the biological functions of PCD.

Except for a significantly hyperactivated level of ferroptosis pathway, patients in cluster 2 showed a significantly higher proportion of ILC subtype, basal patients, and more metastasis (P < 0.001) compared to cluster 5 (**Figure 4C**). Functional enrichments were done between clusters 2 and 5 to explore the putative mechanism altered. PDS of all KEGG pathways was calculated and compared between clusters 2 and 5, among which the adipocytokine signaling pathway, T-cell receptor signaling pathway, TGF-beta signaling pathway, and leukocyte trans-endothelial migration pathway were found hyperactivated in cluster 2, whereas basal transcription factor pathway was significantly suppressed. For further validation, DEGs were calculated (**Figure A3A**) and used as an input for GSEA analyses of both GO and KEGG. The top 40 KEGG pathways and biological functions in GO were shown in **Figures A3B, C**. Intersections of the functional enrichments found immunological functions significantly altered between patients in clusters 2 and 5. In cluster 2, immune-related functions like antigen processing and presentation *via* MHC class Ib (NE: 0.97, NES: 1.46, *P*-value = 0.016), positive regulation of T-cell–mediated cytotoxicity (NE: 0.96, NES: 1.52, *P*-value = 0.010), and regulation of T-cell apoptotic process (NE: 0.93, NES: 1.49, *P*-value = 0.023) were significantly activated. This indicates that patients in cluster 2 are more immune-active compared to patients in cluster 5.

Given the opposed clinical significance of ferroptosis found in clusters 2 and 5, we hypothesized that ferroptosis may have a regulatory function on immunity. Therefore, correlations between ferroptosis pathway and tumor-infiltrating immune cells were explored in both cluster 2 and cluster 5, respectively. In cluster 2, Tgd, iT_reg_, B cell, T_cm_, and macrophage were found significantly correlated with ferroptosis (**Figure 4D**), whereas in cluster 5, significant correlations were seen in NKT, B cell, T_ex_, iT_reg_, T_H_17, nT_reg_, CD8.naive, DC, T_H_1, and Tgd. Among cells that significantly correlated with ferroptosis in both clusters, reversed correlations were seen in Tgd, B cell, and iT_reg_ between the two clusters (Tgd: Rc2 = 0.25, Rc5 = −0.12; B cell: Rc2 = −0.18, Rc5 = 0.34; iT_reg_: Rc2 = −0.23, Rc5 = 0.24). These results suggested that the different regulatory mechanisms of activated and inactivated ferroptosis between cluster 2 and cluster 5 mainly affect the infiltration, but not the function of immune cells.

### Selection of putative biomarker for ferroptosis-activated patients

**Figure 5:**
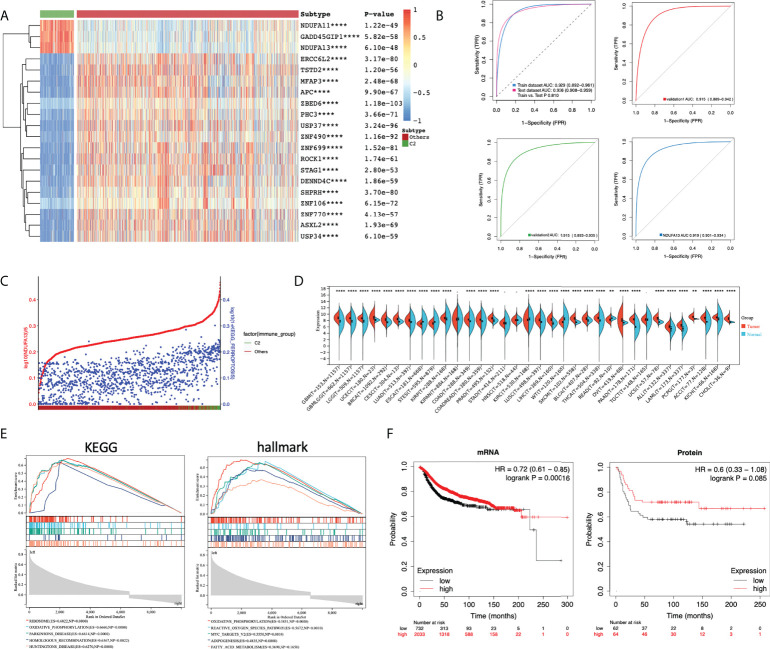
Selection of putative biomarker for cluster 2 patients. **(A)** Heatmap of the top 20 putative biomarkers between cluster 2 and other breast cancer patients. DEGs were calculated using limma; DEGs with adjusted *P*-value < 0.05 and |log2FC| > 1 were selected for ROC analysis. DEGs were ranged according to the AUC and only top 20 genes were illustrated. **(B)** ROC plot comparing the predictive value of the three over-expressed genes as biomarkers of cluster 2 patients. A three-gene index was generated using Lasso regression. The predicted value of the three-gene index generated was estimated with TCGA-BRCA patients divided equally into the training cohort and test cohort. Further validations were done using data from the METABRIC (validation 1) and MSK-IMPACT projects (validation 2). **(C)** Scatter plot showing the expression of NDUFA13 (red) and the PDS of ferroptosis pathway (blue) with patients annotated to cluster 2 or others. Patients were ranged by the expression of NDUFA13. **(D)** Pan-cancer expression analysis of NDUFA13 in 27 cancer types using TCGA PANCAN and GTEx dataset. **(E)** GSEA plots of NDUFA13-related pathways and cancer hallmarks. **(F)** Kaplan–Meier survival plots for NDUFA13 expression in breast cancer. Distant metastasis-free survival (DMFS) was used as an endpoint. A two-sided *P*-value < 0.05 was considered significant. **P < 0.01; ****P < 0.0001.

Given the results presented above, patients in cluster 2 are characterized with the hyperactivated ferroptosis pathway and higher immune infiltration. Additional analyses were done to explore putative biomarker genes between the two clusters. DEGs were calculated between C2 and other patients and analyzed using ROC. Only the top 20 DEGs with the highest AUC were left for further comparison (**Table A5**), among which only three genes were significantly overexpressed in cluster 2 (namely, GADD45GIP1, NDUFA11, and NDUFA13; **Figure 5A**) and may work as putative biomarkers. A three-gene predictive model was generated by Lasso regression among the top 20 DEGs. Intriguingly, the predictive model constructed by Lasso only contains the three over-expressed genes. Evaluation of the three-gene index found stable and consistent predictive efficacy between the training cohort and test cohort [Train AUC: 0.929 (0.892−0.961); Test AUC: 0.936 (0.908−0.959); P = 0.810]. External validations were done in the METABRIC and MSK-IMPACT projects. The alterations of PCD were first quantified in both validation cohorts. Patients were clustered under the same criteria and the cluster 2 subset of patients was identified manually. The predictive efficacy of the three-gene predictive model was presented as ROC, and in both validation cohorts, the three-gene panel showed a good efficacy [validation 1: AUC 0.915 (0.889−0.942); validation 2: AUC 0.915 (0.893−0.935)] (**Figure 5B**). Furthermore, NDUFA13 demonstrated good efficacy compared to the three-gene predictive model (**Figure 5B**, AUC 0.919 (0.901−0.934) P = 0.101), suggesting that NDUFA13 can work as a single-gene biomarker for the selection of cluster 2 patients. Moreover, the correlation between NDUFA13 and ferroptosis pathway alteration was shown in **Figure 5C** with patients ranged by the expression of NDUFA13. Based on the results presented, overexpression of NDUFA13 strongly correlated with the status of ferroptosis (R = 0.53, P < 0.0001).

Given the positive correlation identified in our study, putative biological functions between NDUFA13 and ferroptosis may need further demonstration. Firstly, expression analysis revealed over-expression of NDUFA13 in breast cancer and several other cancers (**Figure 5D**), indicating putative oncogenic functions of NDUFA13 in tumors. Then, functional enrichments were done using GSEA, among which TOP 5 significantly enriched KEGG pathways and cancer hallmarks were shown in **Figure 5E**. Positive regulations of oxidative phosphorylation was enriched in both KEGG and hallmark dataset (KEGG: ES = 0.6660, P = 0; HALLMARK: ES = 0.5831, P = 0). In the hallmark dataset, NDUFA13 was found positively correlated with ROS pathway (ES =0.5672, P = 0.001), adipogenesis (ES = 0.4835, P = 0), and fatty acid metabolism (ES = −0.3690,NES = −1.7, P = 0.1650), which were demonstrated factors associated with the occurrence of ferroptosis, indicating a biological correlation between NDUFA13 and ferroptosis. The KMplot was utilized to assess the prognostic effect of NDUFA13 on both mRNA and protein levels. A total of 2,765 breast cancer cases were available for distant metastasis-free survival (DMFS) analysis by mRNA and 126 cases by protein. Our study showed that the overexpression of NDUFA13 was correlated with a significant increase in the DMFS of breast cancer patients (**Figure 5F**, mRNA: P < 0.001, Protein: P = 0.085).

### Validation of NDUFA13 as putative biomarker for ferroptosis

**Figure 6:**
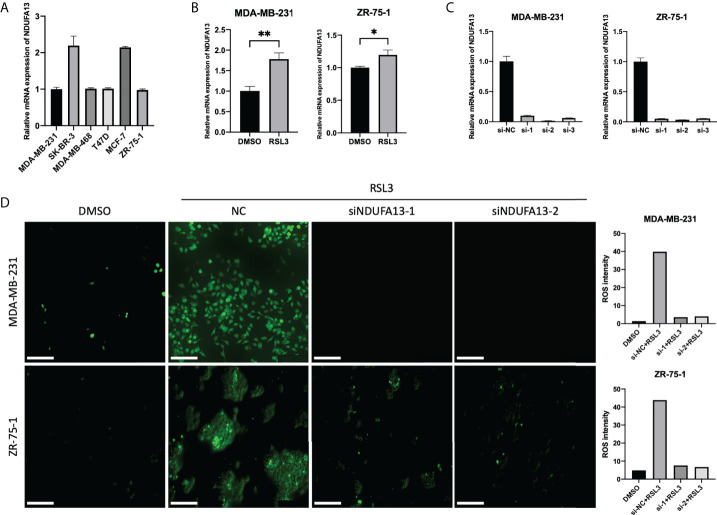
Validations of NDUFA13 as a biomarker for ferroptosis. **(A)** Expression of NDUFA13 in human breast cancer cell lines. **(B)** Expression of NDUFA13 mRNA in human breast cancer cell lines treated with DMSO or RSL3 (10 μM) for 24 h. **(C)** Efficiency test of NDUFA13 siRNA knockdown (KD). **(D)** Intracellular level of reactive oxygen species (ROS) in breast cancer cells treating with DMSO or RSL3 (10 μM) followed by NDUFA13 KD. A two-sided P-value < 0.05 was considered significant. *P < 0.05; **P < 0.01.

To fully validate the correlation between NDUFA13 and ferroptosis, the cellular expression level of NDUFA13 mRNA was first evaluated in order to provide the basic level of expression between subtypes. Comparatively, SK-BR-3 and MCF-7 exhibit a higher level of NDUFA13; nevertheless, a similar expression pattern was seen between cell lines (**Figure 6A**). MDA-MB-231 and ZR-75-1 were selected for further experimental demonstration considering the similar basic expression level and the molecular subtypes. After ferroptosis induced by 24-h incubation of RSL3 (10 μM), both cells showed significantly elevated levels of NDUFA13 compared to DMSO-treated groups (**Figure 6B**, P < 0.01).

We then tested the intracellular level of ROS in MDA-MB-231 and ZR-75-1 after knocking down NDUFA13 (**Figure 6C**). Both cells showed a reduction of ROS in NDUFA13 knockdown (KD) groups compared with NC after treating with RSL3. These results indicated that NDUFA13 had a positive correlation with ferroptosis (**Figure 6D**).

## Discussion

By adopting the bioinformatic algorithm “pathifier”, we quantified five PCD pathways in breast cancer patients, so as to comprehensively analyze the putative biological, pathological, and clinical significance of PCD pathways in breast cancer on a pathway level. Given the results presented in our study, we found significantly activated PCD in cancer patients, among which ferroptosis demonstrated a significant correlation with the progression of breast cancer. Correlation analysis between PCD identified intra-tumor heterogeneity of breast cancer. Therefore, clustering of patients based on the PDS was done. Comparisons between subgroups highlighted specifically activated ferroptosis in cluster 2. Functional enrichment identified the distinct status of immunity and tumor microenvironment between patients with activated and inactivated ferroptosis pathways. To fulfill the clinical significance of ferroptosis, NDUFA13 was identified as a selective biomarker of ferroptosis activation and further demonstrated putative biological functions in the regulation of ferroptosis.

Previous works focusing on the pathway alterations in cancer were first seen in works done by Wang et al. regarding the Hippo signaling pathway (22). A comprehensive PANCAN analysis of 19 Hippo core genes across 33 cancer types using multi-omics data from TCGA was done and a YAP/TAZ transcriptional target signature of 22 genes was developed to characterize Hippo pathway activity. Another research focusing on the oncogenic pathways in human cancer was done by Li et al. (23). By reviewing literature published and further explored with TCGA data, multi-omics features of oncogenic pathways were summarized and restored as an online database. Despite the inspiring work done, all the discussion remained on the gene level, which hardly reflects the status of each pathway. Therefore, we used PDS generated by “pathifier” to reflect the pathway activity and further explore the clinical significance on a pathway level, which demonstrated more accurate efficacy than other methods.

Crosstalk between PCD pathways and immunity was previously summarized in a review done by Tang et al. (2), in which cell death was categorized into two kinds: immunogenic cell death (ICD) that alerts and triggers immunity against dead-cell antigens, including ferroptosis, necroptosis, pyroptosis, and autophagic cell death and tolerogenic cell death (TCD) that actively inhibits immune responses (24–27). Apoptosis was considered TCD mostly but grew an importance in the activation of tumor immunity under certain conditions. The correlation between the ferroptosis pathway and tumor immunity identified in our study was previously demonstrated on a cellular level. Recent results done by Wang et al. reveal that CD8^+^ T cells drive ferroptosis in tumor cells and the tumor suppressive function of interferon (IFN)-gamma secreted by CD8^+^ T cells in response to immune checkpoint blockade meditated by ferroptosis, suggesting that the immune system may function in part through ferroptosis to prevent tumorigenesis, and ferroptosis may hold the key to tumor immunotherapy response (28, 29). Despite numerous works done, the immunological function of ferroptosis remained underestimated considering the result presented in our study. Intra-tumor heterogeneity contributes to the differential alteration of tumor immunity caused by ferroptosis. Here, we characterized a subset of breast cancer patients with hyperactivated status of ferroptosis and necroptosis pathway and overexpressed NDUFA13, in which activation of ferroptosis promoting the infiltration of anti-tumor cells like Tgd, therefore, might correlate with better responsiveness of immunotherapy in breast cancer. Previously reported biological functions of NDUFA13 mainly involved in the IFN/all-trans-retinoic acid (IFN/RA)–induced cell death and transfer of electrons from NADH to the respiratory chain, therefore, impacted the mitochondrial and cellular ROS production.

In conclusion, we quantified the status alteration of PCD pathways and highlighted the significant correlation of ferroptosis with early recurrence and progression of breast cancer. Intra-tumor heterogeneity of breast cancer was detected based on the status of the ferroptosis pathway. Mechanism analyses further revealed distinct tumor microenvironment and immunological function of ferroptosis between patients. NDUFA13 expression was identified as a positive biomarker for ferroptosis pathway activation in breast cancer patients.

## Data availability statement

The original contributions presented in the study are included in the article/**Supplementary Material**. Further inquiries can be directed to the corresponding authors.

## Author contributions

YYL and TL analyzed and interpreted the data and drafted the work. YYL and TL were major contributors in writing the manuscript. XK revised the manuscript. DZ helped with software used for analyses. NS was granted the funding. NS and YYL designed and corresponded for the work. All authors read and approved the final manuscript.

## Funding

This research is funded by the Beijing Science And Technology Innovation Medical Development Foundation (KC2021-JX-0044-2) and Guangdong Health Management Society - Breast Disease Professional Committee Fund (2021001).

## Conflict of interest

The authors declare that the research was conducted in the absence of any commercial or financial relationships that could be construed as a potential conflict of interest.

## Publisher’s note

All claims expressed in this article are solely those of the authors and do not necessarily represent those of their affiliated organizations, or those of the publisher, the editors and the reviewers. Any product that may be evaluated in this article, or claim that may be made by its manufacturer, is not guaranteed or endorsed by the publisher.
