# Supplementary material for: Quantification of ferroptosis pathway status revealed heterogeneity in breast cancer patients with distinct immune microenvironment
Source: Front Oncol. 2022 Sep 2;12:956999. doi: 10.3389/fonc.2022.956999 (PMC9478851; doi:10.3389/fonc.2022.956999)
Supplement: Supplementary file 1 [file DataSheet_1.pdf]

Appendices

1 Appendice Figures

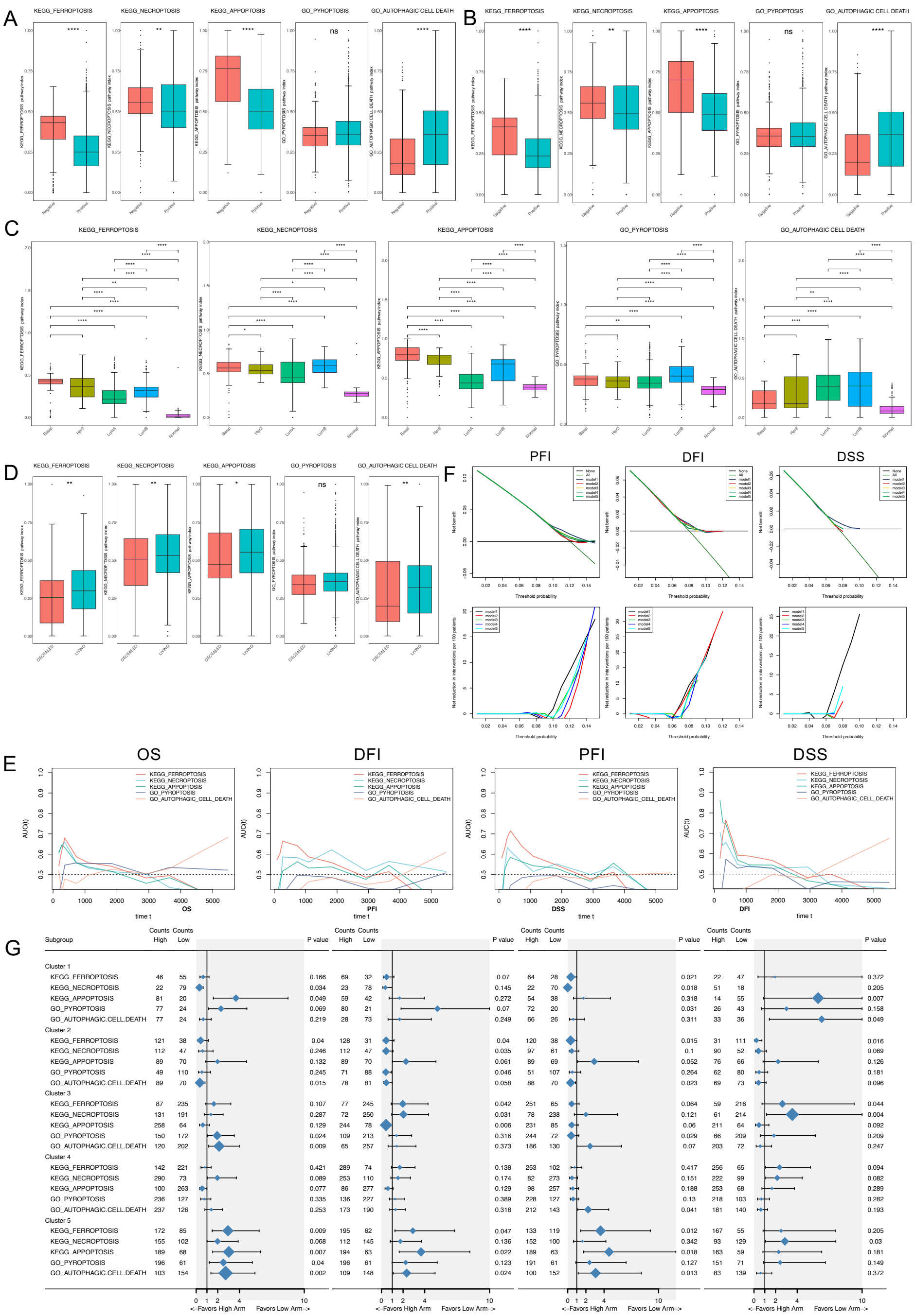

Appendice Figure A1. Clinical characteristics and prognostic significance of programmed cell death pathway alterations in breast cancer. Comparisons of PDS were done between different **A.** status of ER; **B.** status of PR; **C.** PAM50 subtypes; **D.** vital status; **E.** DCA of the PDS of programmed cell death pathways as prognostic models for clinical decision making regarding 3 years

PFI, DFI and DSS. Model 1: KEGG_FERROPTOSIS; model 2: KEGG_NECROPTOSIS; model 3: KEGG_APPOPTOSIS; model 4: GO_PYROPTOSIS; model 5: GO_AUTOPHAGIC_CELL_DEATH. **F.** TimeROC plots of programmed cell death pathways in breast cancer regarding OS, PFI, DFI and DSS. Time point used includes 0, 90, 180, 270, 365, 730, 1095, 1825, 2920, 3650, 5475 days. **G.** Survival analyses of programmed cell death pathways in breast cancer regarding overall survival (OS), progression-free interval (PFI), disease-free interval (DFI) and disease-specific survival (DSS). Hazard ratio (HR) and 95% confidence interval (95%CI) was shown in the forest plot. *P < 0.05; **P < 0.01; ***P < 0.001; ****P < 0.0001.

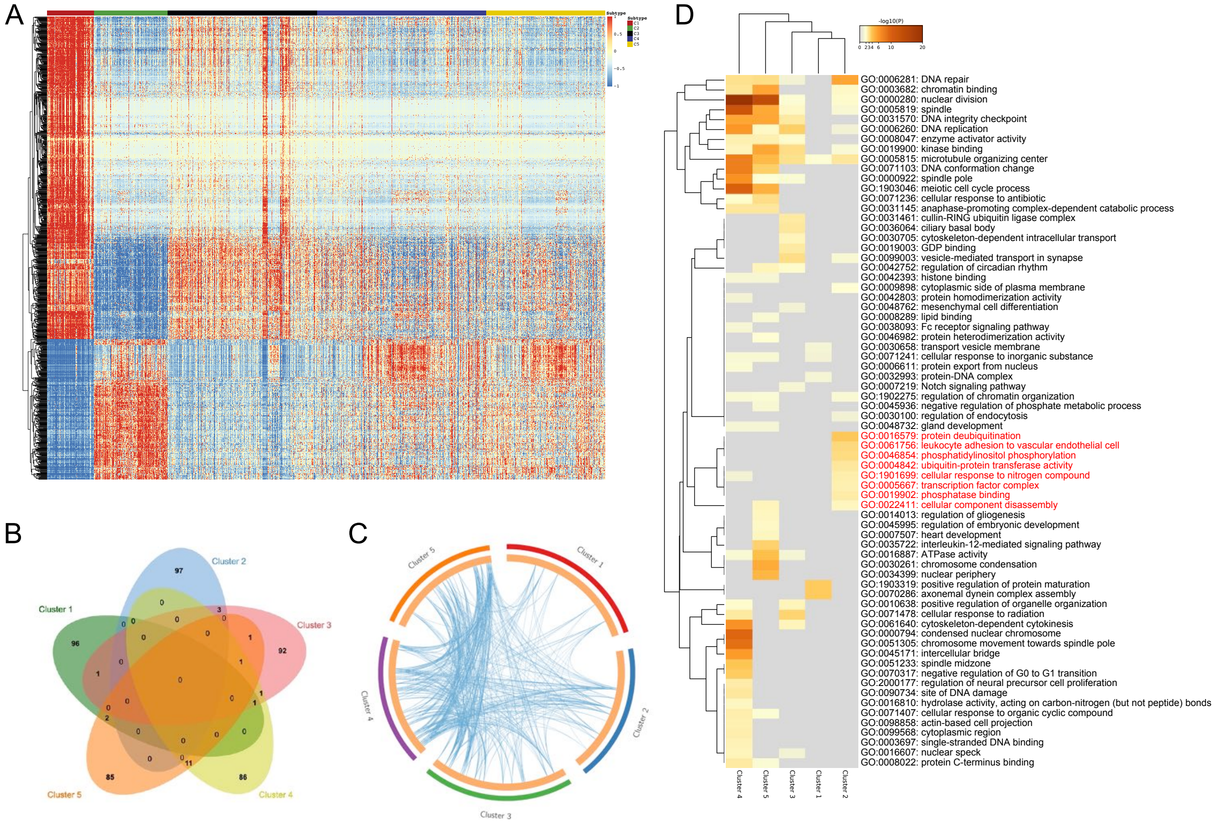

Appendice Figure A2. Differential analysis and functional enrichment between clusters. A. Heatmap of the top 1000 differentially expressed genes (DEGs) between clusters. DEGs were calculated using limma with adjust P value < 0.05 and |log2FC| > 1 used for selection. DEGs were ranged according to the AUC and top 1000 genes were clustered and illustrated; **B.** Venn plot of top 100 DEGs in each cluster; **C.** Specific DEGs of each cluster were put into multi-group functional enrichments. Overlap between the shared terms were illustrated as circos plot where blue curves link genes that belong to the same enriched ontology term. The inner circle represents gene lists, where hits are arranged along the arc. Genes that hit multiple lists are colored in dark orange, and genes

unique to a list are shown in light orange. **D.** Heatmap of enriched terms across clusters, colored by p-values.

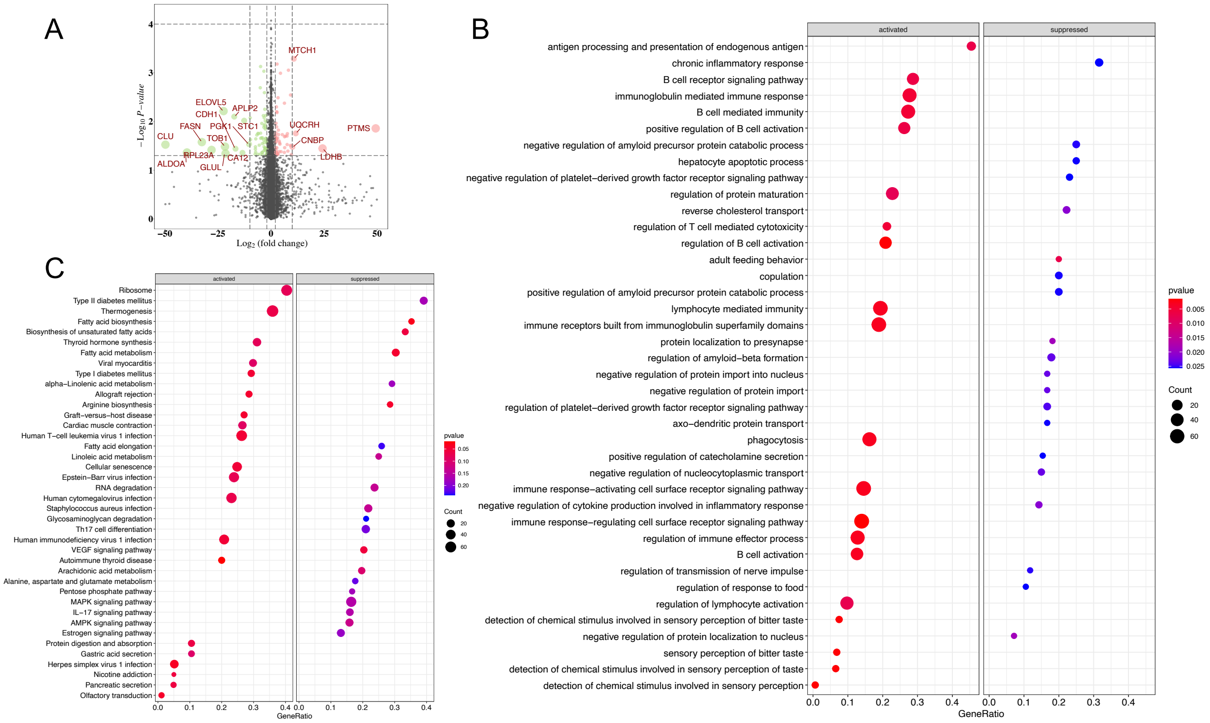

Appendice Figure A3. Functional enrichment between cluster 2 and cluster 5. A. Volcano plot of differential expressed genes between cluster 2 and cluster 5; Bubble plot of top 40 **B.** GO or **C.** KEGG enrichment using GSEA were shown. Items were divided into categories of activation or suppression.

2 Appendice Tables

Appendice Table A1. Comparisons of PDS between clusters.

	Tukey's multiple comparisons test			one-way ANOVA
	Mean	95.00% CI	Adjusted P Value	P value
KEGG_FERROPTOSIS				<0.0001
C1 vs. C2	-0.5413	-0.5804 to -0.5023	<0.0001	
C1 vs. C3	-0.162	-0.1970 to -0.1270	<0.0001	
C1 vs. C4	-0.3038	-0.3383 to -0.2692	<0.0001	
C1 vs. C5	-0.2704	-0.3064 to -0.2343	<0.0001	
C2 vs. C3	0.3794	0.3496 to 0.4091	<0.0001	
C2 vs. C4	0.2376	0.2084 to 0.2668	<0.0001	
C2 vs. C5	0.271	0.2400 to 0.3020	<0.0001	
C3 vs. C4	-0.1418	-0.1653 to -0.1183	<0.0001	
C3 vs. C5	-0.1084	-0.1341 to -0.08274	<0.0001	
C4 vs. C5	0.03339	0.008373 to 0.05841	0.0026	
KEGG_NECROPTOSIS				<0.0001
C1 vs. C2	-0.5536	-0.5899 to -0.5174	<0.0001	
C1 vs. C3	-0.1855	-0.2180 to -0.1531	<0.0001	
C1 vs. C4	-0.2789	-0.3109 to -0.2469	<0.0001	
C1 vs. C5	-0.3787	-0.4122 to -0.3453	<0.0001	
C2 vs. C3	0.3681	0.3405 to 0.3957	<0.0001	
C2 vs. C4	0.2747	0.2476 to 0.3018	<0.0001	
C2 vs. C5	0.1749	0.1462 to 0.2036	<0.0001	

C3 vs. C4	-0.0934	-0.1152 to -0.07164	<0.0001	
C3 vs. C5	-0.1932	-0.2170 to -0.1694	<0.0001	
C4 vs. C5	-0.0998	-0.1230 to -0.07660	<0.0001	
KEGG_APPOPTOSIS				<0.0001
C1 vs. C2	-0.2432	-0.2926 to -0.1938	<0.0001	
C1 vs. C3	-0.02287	-0.06710 to 0.02137	0.6198	
C1 vs. C4	-0.268	-0.3116 to -0.2244	<0.0001	
C1 vs. C5	-0.318	-0.3636 to -0.2724	<0.0001	
C2 vs. C3	0.2203	0.1827 to 0.2579	<0.0001	
C2 vs. C4	-0.0248	-0.06169 to 0.01208	0.3527	
C2 vs. C5	-0.0748	-0.1140 to -0.03565	<0.0001	
C3 vs. C4	-0.2451	-0.2748 to -0.2155	<0.0001	
C3 vs. C5	-0.2951	-0.3276 to -0.2627	<0.0001	
C4 vs. C5	-0.05	-0.08161 to -0.01838	0.0002	
GO_PYROPTOSIS				<0.0001
C1 vs. C2	-0.1341	-0.1816 to -0.08652	<0.0001	
C1 vs. C3	-0.1007	-0.1433 to -0.05810	<0.0001	
C1 vs. C4	-0.1139	-0.1559 to -0.07188	<0.0001	
C1 vs. C5	-0.1588	-0.2026 to -0.1149	<0.0001	
C2 vs. C3	0.03337	-0.002819 to 0.06956	0.0871	
C2 vs. C4	0.02017	-0.01535 to 0.05568	0.5291	
C2 vs. C5	-0.02472	-0.06241 to 0.01297	0.3788	
C3 vs. C4	-0.0132	-0.04176 to 0.01535	0.7138	

C3 vs. C5	-0.05809	-0.08931 to -0.02686	<0.0001	
C4 vs. C5	-0.04488	-0.07532 to -0.01445	0.0006	
GO_AUTOPHAGIC CELL DEATH				<0.0001
C1 vs. C2	-0.184	-0.2281 to -0.1400	<0.0001	
C1 vs. C3	-0.4082	-0.4477 to -0.3688	<0.0001	
C1 vs. C4	-0.0789	-0.1178 to -0.03996	<0.0001	
C1 vs. C5	-0.4071	-0.4478 to -0.3664	<0.0001	
C2 vs. C3	-0.2242	-0.2577 to -0.1906	<0.0001	
C2 vs. C4	0.1051	0.07223 to 0.1381	<0.0001	
C2 vs. C5	-0.223	-0.2580 to -0.1881	<0.0001	
C3 vs. C4	0.3293	0.3029 to 0.3558	<0.0001	
C3 vs. C5	0.001141	-0.02780 to 0.03008	1	
C4 vs. C5	-0.3282	-0.3564 to -0.3000	<0.0001	

Appendice Table A2. Comparisons of clinical characteristics between clusters.

level	C1 (n=101)	C2 (n=159)	C3 (n=323)	C4 (n=364)	C5 (n=257)	P value
Age [mean (SD)]	57.70 (14.52)	59.38 (13.75)	59.39 (12.74)	57.92 (13.31)	57.29 (13.33)	0.276
Gender (%)						0.823
FEMALE	100 (99.0)	156 (98.1)	320 (99.1)	360 (98.9)	254 (98.8)	
MALE	1 (1.0)	3 (1.9)	2 (0.6)	4 (1.1)	3 (1.2)	
Sample_type (%)						<0.001
Primary Tumor	13 (12.9)	157 (98.7)	304 (94.1)	361 (99.2)	256 (99.6)	
Solid Tissue Normal	88 (87.1)	2 (1.3)	19 (5.9)	3 (0.8)	1 (0.4)	
Histological_type (%)						<0.001
Infiltrating Carcinoma NOS	0 (0.0)	1 (0.6)	0 (0.0)	0 (0.0)	0 (0.0)	
Infiltrating Ductal Carcinoma	3 (2.7)	102 (64.2)	198 (61.3)	295 (81.0)	200 (77.8)	
Infiltrating Lobular Carcinoma	7 (6.3)	38 (23.9)	89 (27.6)	36 (9.9)	32 (12.5)	
Medullary Carcinoma	2 (1.8)	0 (0.0)	0 (0.0)	5 (1.4)	1 (0.4)	
Metaplastic Carcinoma	2 (1.8)	4 (2.5)	0 (0.0)	1 (0.3)	2 (0.8)	
Mixed Histology	0 (0.0)	4 (2.5)	10 (3.1)	8 (2.2)	7 (2.7)	
Mucinous Carcinoma	1 (1.0)	3 (1.9)	5 (1.5)	5 (1.4)	4 (1.6)	
Other	96 (86.4)	7 (4.4)	20 (6.2)	13 (3.6)	11 (4.3)	
T (%)						0.352
T1	26 (25.7)	34 (21.4)	103 (31.9)	91 (25.0)	54 (21.0)	
T2	58 (57.4)	93 (58.5)	169 (52.3)	218 (59.9)	158 (61.5)	
T3	11 (10.9)	26 (16.4)	41 (12.7)	38 (10.4)	33 (12.8)	

T4	6 (5.9)	5 (3.1)	9 (2.8)	16 (4.4)	11 (4.3)	
TX	0 (0.0)	1 (0.6)	0 (0.0)	1 (0.3)	1 (0.4)	
N (%)						0.333
N0	48 (47.5)	77 (48.4)	156 (48.3)	168 (46.2)	110 (42.8)	
N1	35 (34.7)	48 (30.2)	120 (37.2)	120 (33.0)	86 (33.5)	
N2	11 (10.9)	14 (8.8)	24 (7.4)	46 (12.6)	37 (14.4)	
N3	4 (4.0)	14 (8.8)	17 (5.3)	26 (7.1)	19 (7.4)	
NX	3 (3.0)	6 (3.8)	5 (1.5)	4 (1.1)	5 (1.9)	
M (%)						<0.001
M0	88 (87.1)	107 (67.3)	276 (85.4)	319 (87.6)	221 (86.0)	
M1	3 (3.0)	5 (3.1)	4 (1.2)	6 (1.6)	6 (2.3)	
MX	10 (9.9)	47 (29.6)	42 (13.0)	39 (10.7)	30 (11.7)	
ER (%)						<0.001
Indeterminate	1 (1.0)	1 (0.6)	0 (0.0)	0 (0.0)	1 (0.4)	
Negative	20 (19.8)	51 (32.1)	15 (4.6)	128 (35.2)	44 (17.1)	
Positive	68 (67.3)	100 (62.9)	296 (91.6)	223 (61.3)	195 (75.9)	
PR (%)						<0.001
Indeterminate	1 (1.0)	0 (0.0)	0 (0.0)	2 (0.5)	2 (0.8)	
Negative	30 (29.7)	67 (42.1)	45 (13.9)	157 (43.1)	75 (29.2)	
Positive	58 (57.4)	85 (53.5)	266 (82.4)	191 (52.5)	163 (63.4)	
HER2 (%)						0.003
Equivocal	7 (6.9)	33 (20.8)	56 (17.3)	47 (12.9)	44 (17.1)	
Indeterminate	0 (0.0)	3 (1.9)	2 (0.6)	4 (1.1)	3 (1.2)	

Negative	50 (49.5)	68 (42.8)	183 (56.7)	187 (51.4)	127 (49.4)	
Positive	21 (20.8)	20 (12.6)	32 (9.9)	70 (19.2)	44 (17.1)	
PAM50 (%)						<0.001
Basal	4 (4.0)	19 (11.9)	4 (1.2)	86 (23.6)	31 (12.1)	
Her2	0 (0.0)	8 (5.0)	0 (0.0)	39 (10.7)	20 (7.8)	
LumA	15 (14.9)	21 (13.2)	226 (70.0)	110 (30.2)	72 (28.0)	
LumB	0 (0.0)	10 (6.3)	29 (9.0)	74 (20.3)	82 (31.9)	
Normal	75 (74.3)	1 (0.6)	16 (5.0)	1 (0.3)	1 (0.4)	
Margin_status (%)						<0.001
Close	3 (3.0)	6 (3.8)	10 (3.1)	6 (1.6)	7 (2.7)	
Negative	71 (70.3)	134 (84.3)	263 (81.4)	315 (86.5)	216 (84.0)	
Positive	3 (3.0)	13 (8.2)	25 (7.7)	20 (5.5)	20 (7.8)	
Menopause_status (%)						<0.001
Peri	3 (3.0)	9 (5.7)	18 (5.6)	25 (6.9)	21 (8.2)	
Post	49 (48.5)	102 (64.2)	212 (65.6)	235 (64.6)	160 (62.3)	
Pre	24 (23.8)	32 (20.1)	67 (20.7)	80 (22.0)	51 (19.8)	
Vital_status (%)						<0.001
DECEASED	41 (40.6)	21 (13.2)	45 (13.9)	54 (14.8)	35 (13.6)	
LIVING	60 (59.4)	138 (86.8)	277 (85.8)	310 (85.2)	222 (86.4)	
Cancer_status (%)						0.019
TUMOR FREE	73 (72.3)	133 (83.6)	278 (86.1)	304 (83.5)	223 (86.8)	
WITH TUMOR	18 (17.8)	22 (13.8)	33 (10.2)	45 (12.4)	28 (10.9)	
Neoadjuvant_treatment (%)						0.93

No	100 (99.0)	158 (99.4)	317 (98.1)	360 (98.9)	253 (98.4)	
Yes	1 (1.0)	1 (0.6)	5 (1.5)	4 (1.1)	3 (1.2)	
Targeted_therapy (%)						<0.001
NO	1 (1.0)	3 (1.9)	16 (5.0)	18 (4.9)	9 (3.5)	
YES	46 (45.5)	51 (32.1)	161 (49.8)	197 (54.1)	128 (49.8)	

Appendice Table A3. Biomarkers for each cluster.

Cluster 1	Cluster 2	Cluster 3	Cluster 4	Cluster 5
COX6C	RAPH1	RP11-303E16.2	CDKN1B	GNG12-AS1
RHBDL1	ZBED6	CMC2	TUBA1C	ARHGAP20
KREMEN2	NBEAL1	NUTF2	SHCBP1	TIMELESS
TIGD3	PWAR5	CENPW	RRM2	H2AFY
WBSCR28	USP37	PDCD5	ANLN	ESPL1
HAGHL	CTC-339F2.2	SPG11	CALCOCO1	ZWINT
SYNGR3	ZNF490	PDHA1	FAM83D	ARL6IP1
PACSIN1	CRYBG3	ORC6	FAM47E	HNRNPAB
ARTN	ZNF699	IL6ST	RP11-180M15.7	MIR99AHG
HIST2H4A	PCNX1	AURKB	EZH1	CARMN
HES6	ERCC6L2	FAM214A	BUB1	RP5-965F6.2
CNTD2	SHPRH	TBC1D9	ACTR3	ADAMTS9-AS2
CYSRT1	PLEKHM3	RBM43	EPB41L4A-AS1	RP11-421P23.2
PKMYT1	REL	PJA2	RP3-414A15.12	RP11-1024P17.1
FAM72D	TCP11L2	THSD4	RP4-635E18.8	ANGPTL1
TFR2	RP11-701H24.3	FAM96B	KPNA2	ADAMTS5
C9orf172	ANAPC1	CDT1	ERCC6L	RP11-679B19.1
SPEF1	CTD-2647L4.4	FBXO38	DLGAP5	CCNB1
PLAC1	RP11-777F6.3	MYO5C	CEP55	RBMS3
CNIH2	KLHL11	RAB27B	EXO1	CAV2
LINC00337	RBMS2P1	CA12	KLHDC1	LMNB1
DSCAM-AS1	ZNF81	TGOLN2	PGK1	MAGI2-AS3
LAGE3	ZNF106	CCNE1	BUB1B	UHRF1
C16orf59	RP4-791C19.1	CDCA3	CIRBP	TMEM106C
BMPR1B-AS1	GALNT4	RP11-539I5.1	TNS2	HNRNPA2B1
DNASE1L2	PHC3	SHFM1	CKAP2L	RP11-175K6.1

SPARCL1	BMP2K	ECE2	MCM10	FLRT2
NAXE	ZNF221	CCNG2	MKI67	CCDC36
TIMM17A	ASXL2	CHIC1	ELMOD3	MAZ
FOXD3-AS1	RP11-399B17.1	POLK	MTHFD2	RACGAP1
C6orf99	MAP2K4P1	CDC25A	SMC4	CCNE2
FLAD1	NHSL2	FBXL17	CENPI	KIF22
LRRC73	ZDHHC20P4	CENPA	RP11-348N5.9	CDC25C
MRPL13	KIAA1109	EMC8	SCRN2	FKBP4
ZMYND10	APC	FAM179B	AURKA	CCNF
CCDC78	SETX	TP53BP1	RP11-180M15.6	EBF1
POLR3K	LL0XNC01-237H1.2	HBP1	RP11-2E11.9	CKS2
C9orf116	AC000123.2	APH1B	DEPDC1	KIFC1
BOLA2B	CREB1	CREBRF	GTF2IRD2	VEGFD
F12	PIK3CG	CREBL2	HJURP	TOP2A
CACYBP	EEA1	MAP3K1	CLSPN	ABCA8
SCAMP3	RP11-815J21.4	FAM114A2	CDCA8	RUNX1T1
CFAP45	AKAP2	UTP14C	RAPGEF3	ATAD2
GRM4	ZNF852	FAM64A	CCNB2	CDK1
RP5-963E22.6	TRIP12	FCHO2	CENPF	ADAMTS9-AS1
DNAAF3	ALG11	SLC24A1	FAM111B	FREM1
GPRIN1	AC094019.4	LZTFL1	MIR497HG	P2RY12
RDH16	GPATCH2L	SNAP23	RP11-571L19.8	KIF11
ATP5G1P4	UBE4A	JADE2	GTSE1	F3
VPS72	REV3L	ANKRA2	GPIHBP1	SCARA5
CAPSL	SCAF11	APPL2	MELK	AP000473.8
CLPSL2	USF3	TCF12	ACBD4	RP11-175K6.2
IBSP	RFX7	FYCO1	RAD51	SLC16A7
RIPPLY3	CPED1	GIGYF2	NCAPH	GRHL2
RIIAD1	GIT2	FAM172A	DHRS12	LRRN4CL

DAP3	ROCK1	MRPS5	SIRT3	PCNA
ITPKA	RN7SKP74	RALGPS2	KNL1	HNRNPK
PPP2R2C	RP11-88I18.2	AFF1	STIL	MAD2L1
DNAJC1	ATRX	CKS1B	ADAM33	AP000473.5
RNF187	ACER2	CPEB2	KIAA1524	CAV1
TNFRSF18	VPS13C	FOXA1	CRY2	NCAPG
TPSP2	RP11-701H24.8	EFCAB14	ASPM	LYVE1
RAB26	KB-318B8.7	CIPC	SGO1	PGM5P4
PRR19	COPE	FBXL3	RP11-96C23.13	RP11-3B7.1
GPR19	RIF1	FRY	PALM	STARD9
CCDC151	RP11-87H9.5	BBS4	PSME4	MAB21L1
HTR1D	BDP1	NUDT1	ECT2	NAALAD2
HCN2	SUCLG2-AS1	RASEF	PLIN5	ALDH1A2
ENTPD8	RP1-253P7.4	CDC45	KIF23	HMMR
FAM72A	ZNF510	TSTD2	CBX7	ABCA9
KISS1R	PPP1R35	INPP4B	CHEK1	ASPA
H2AFZ	NFAT5	CACNA1D	GTF2IRD2B	RASSF9
PARP1	FKBP2	COL4A3BP	LINC01402	PBK
C1orf43	CEP350	FAM13B	LYRM9	PPP5C
LSM4	SMG1	DNAJC16	GCH1	TMEM132C
IGFALS	PTPN4	KIF13B	SLC7A5	EBF2
CACHD1	USP34	PIF1	CENPE	CNTNAP3P2
AMOTL1	MKLN1	TRIM23	OIP5	PEAR1
JTB	ZNF148	CSNK1A1	ACTR2	PREX2
MIR568	SBF2	RANBP1	CTD-2510F5.4	CNTNAP3B
E2F1	STX17	CASC4	ARHGAP11A	ACVR1C
FAM188B2	C9orf142	PAPD4	USB1	LDB2
MCRIP2	DMXL1	UQCRH	CALU	RP11-823E8.3
CAMP	PIKFYVE	DDX39A	ORC1	HNRNPF

AC009005.2	ZNF805	NBR1	KIF2C	UBE2T
NUF2	RP11-447D11.3	PGGT1B	SRSF5	
HGH1	ASH1L	LINC00504		
KIAA0101	SSNA1	CTD-2555O16.2		
CTB-49A3.4	TMEM245	ZNF619		
KRT18P10	GADD45GIP1	NUDT12		
DMD	DRAP1	STAM2		
SIAH2	TRAF6	HAUS8		
FAM189B	FER			
ATP2A1-AS1	FAM175A			
NUAK2	CCDC124			
RP11-424C20.2	KIF2A			
	BIRC6			

Appendice Table A4. Correlations of programmed cell death pathways and the seven-step Cancer-Immunity Cycle in all samples.

gene	immune_cells	cor	p.value
KEGG_FERROPTOSIS	Step1..release.of.cancer.cell.antigens	0.099280321	0.000569927
KEGG_FERROPTOSIS	Step2..cancer.antigen.presentation	0.021742911	0.45156129
KEGG_FERROPTOSIS	Step3..priming.and.activation	0.199695284	2.87E-12
KEGG_FERROPTOSIS	T.cell.recruiting	0.071534241	0.013151259
KEGG_FERROPTOSIS	CD4.T.cell.recruiting	-0.142201912	7.49E-07
KEGG_FERROPTOSIS	CD8.T.cell.recruiting	0.081661869	0.004628501
KEGG_FERROPTOSIS	Th1.cell.recruiting	0.182097296	2.05E-10
KEGG_FERROPTOSIS	Dendritic.cell.recruiting	0.03722864	0.197301237
KEGG_FERROPTOSIS	Th22.cell.recruiting	0.082620796	0.004167739
KEGG_FERROPTOSIS	Macrophage.recruiting	-0.023168961	0.422435946
KEGG_FERROPTOSIS	Monocyte.recruiting	-0.118577945	3.79E-05
KEGG_FERROPTOSIS	Neutrophil.recruiting	-0.137578278	1.70E-06
KEGG_FERROPTOSIS	NK.cell.recruiting	0.154619692	7.23E-08
KEGG_FERROPTOSIS	Eosinophil.recruiting	0.214866734	5.21E-14
KEGG_FERROPTOSIS	Basophil.recruiting	0.082965495	0.004012545
KEGG_FERROPTOSIS	Th17.cell.recruiting	-0.066583037	0.0210201
KEGG_FERROPTOSIS	B.cell.recruiting	0.076626685	0.007891582
KEGG_FERROPTOSIS	Th2.cell.recruiting	0.094837579	0.000999614
KEGG_FERROPTOSIS	Treg.cell.recruiting	-0.153582817	8.86E-08
KEGG_FERROPTOSIS	MDSC.recruiting	-0.160213208	2.37E-08
KEGG_FERROPTOSIS	Step5..infiltration.of.immune.cells.into.tumors	-0.174719419	1.09E-09

KEGG_FERROPTOSIS	Step6..recognition.of.cancer.cells.by.T.cells	0.066351188	0.02147259
KEGG_FERROPTOSIS	Step7..killing.of.cancer.cells	0.091598816	0.001484099
KEGG_NECROPTOSIS	Step1..release.of.cancer.cell.antigens	0.060523772	0.035974367
KEGG_NECROPTOSIS	Step2..cancer.antigen.presentation	0.087601658	0.002377146
KEGG_NECROPTOSIS	Step3..priming.and.activation	0.207462984	3.83E-13
KEGG_NECROPTOSIS	T.cell.recruiting	-0.028470019	0.324224561
KEGG_NECROPTOSIS	CD4.T.cell.recruiting	-0.21798523	2.20E-14
KEGG_NECROPTOSIS	CD8.T.cell.recruiting	-0.029125831	0.313199328
KEGG_NECROPTOSIS	Th1.cell.recruiting	0.073844287	0.010469095
KEGG_NECROPTOSIS	Dendritic.cell.recruiting	-0.069755162	0.0156136
KEGG_NECROPTOSIS	Th22.cell.recruiting	0.02050882	0.477656437
KEGG_NECROPTOSIS	Macrophage.recruiting	-0.138090816	1.56E-06
KEGG_NECROPTOSIS	Monocyte.recruiting	-0.192776188	1.62E-11
KEGG_NECROPTOSIS	Neutrophil.recruiting	-0.238459371	5.46E-17
KEGG_NECROPTOSIS	NK.cell.recruiting	0.041637225	0.149279952
KEGG_NECROPTOSIS	Eosinophil.recruiting	0.151331033	1.37E-07
KEGG_NECROPTOSIS	Basophil.recruiting	0.011014598	0.702958465
KEGG_NECROPTOSIS	Th17.cell.recruiting	-0.12688702	1.03E-05
KEGG_NECROPTOSIS	B.cell.recruiting	0.092495201	0.001331947
KEGG_NECROPTOSIS	Th2.cell.recruiting	0.079231799	0.00600929
KEGG_NECROPTOSIS	Treg.cell.recruiting	-0.195581463	8.08E-12
KEGG_NECROPTOSIS	MDSC.recruiting	-0.305310464	2.50E-27
KEGG_NECROPTOSIS	Step5..infiltration.of.immune.cells.into.tumors	-0.112121699	9.87E-05

KEGG_NECROPTOSIS	Step6..recognition.of.cancer.cells.by.T.cells	0.091448944	0.001511047
KEGG_NECROPTOSIS	Step7..killing.of.cancer.cells	0.059030414	0.040817515
KEGG_APPOPTOSIS	Step1..release.of.cancer.cell.antigens	0.19173085	2.09E-11
KEGG_APPOPTOSIS	Step2..cancer.antigen.presentation	-0.014761882	0.609298715
KEGG_APPOPTOSIS	Step3..priming.and.activation	0.298857009	3.35E-26
KEGG_APPOPTOSIS	T.cell.recruiting	0.255434685	2.42E-19
KEGG_APPOPTOSIS	CD4.T.cell.recruiting	0.09401739	0.001106098
KEGG_APPOPTOSIS	CD8.T.cell.recruiting	0.28313638	1.41E-23
KEGG_APPOPTOSIS	Th1.cell.recruiting	0.315574093	3.53E-29
KEGG_APPOPTOSIS	Dendritic.cell.recruiting	0.152478818	1.10E-07
KEGG_APPOPTOSIS	Th22.cell.recruiting	0.187883271	5.28E-11
KEGG_APPOPTOSIS	Macrophage.recruiting	0.182036318	2.08E-10
KEGG_APPOPTOSIS	Monocyte.recruiting	0.081744345	0.004587133
KEGG_APPOPTOSIS	Neutrophil.recruiting	0.131616698	4.73E-06
KEGG_APPOPTOSIS	NK.cell.recruiting	0.33411063	1.04E-32
KEGG_APPOPTOSIS	Eosinophil.recruiting	0.255074814	2.72E-19
KEGG_APPOPTOSIS	Basophil.recruiting	0.159329833	2.83E-08
KEGG_APPOPTOSIS	Th17.cell.recruiting	0.079582404	0.005789529
KEGG_APPOPTOSIS	B.cell.recruiting	0.108912819	0.000155732
KEGG_APPOPTOSIS	Th2.cell.recruiting	0.220702953	1.02E-14
KEGG_APPOPTOSIS	Treg.cell.recruiting	-0.019035222	0.509865635
KEGG_APPOPTOSIS	MDSC.recruiting	0.078646212	0.006393123
KEGG_APPOPTOSIS	Step5..infiltration.of.immune.cells.into.tumors	-0.281788715	2.33E-23

KEGG_APPOPTOSIS	Step6..recognition.of.cancer.cells.by.T.cells	0.02895109	0.316112627
KEGG_APPOPTOSIS	Step7..killing.of.cancer.cells	0.115227653	6.27E-05
GO_PYROPTOSIS	Step1..release.of.cancer.cell.antigens	0.090309578	0.0017311
GO_PYROPTOSIS	Step2..cancer.antigen.presentation	0.05876539	0.04173228
GO_PYROPTOSIS	Step3..priming.and.activation	0.168082482	4.61E-09
GO_PYROPTOSIS	T.cell.recruiting	-0.026961533	0.350530523
GO_PYROPTOSIS	CD4.T.cell.recruiting	-0.093760706	0.001141517
GO_PYROPTOSIS	CD8.T.cell.recruiting	0.062384638	0.030631886
GO_PYROPTOSIS	Th1.cell.recruiting	0.119568812	3.26E-05
GO_PYROPTOSIS	Dendritic.cell.recruiting	-0.04176474	0.14803709
GO_PYROPTOSIS	Th22.cell.recruiting	0.053334111	0.064644604
GO_PYROPTOSIS	Macrophage.recruiting	-0.106969017	0.000204122
GO_PYROPTOSIS	Monocyte.recruiting	-0.09121633	0.001553764
GO_PYROPTOSIS	Neutrophil.recruiting	-0.191652641	2.13E-11
GO_PYROPTOSIS	NK.cell.recruiting	0.096943153	0.000768115
GO_PYROPTOSIS	Eosinophil.recruiting	-0.115924797	5.65E-05
GO_PYROPTOSIS	Basophil.recruiting	-0.191602368	2.15E-11
GO_PYROPTOSIS	Th17.cell.recruiting	-0.052594906	0.068445401
GO_PYROPTOSIS	B.cell.recruiting	0.018844354	0.514119033
GO_PYROPTOSIS	Th2.cell.recruiting	0.049867266	0.084087302
GO_PYROPTOSIS	Treg.cell.recruiting	-0.093220091	0.001219564
GO_PYROPTOSIS	MDSC.recruiting	-0.150952232	1.47E-07
GO_PYROPTOSIS	Step5..infiltration.of.immune.cells.into.tumors	-0.168166428	4.53E-09

GO_PYROPTOSIS	Step6..recognition.of.cancer.cells.by.T.cells	-0.011637572	0.68702257
GO_PYROPTOSIS	Step7..killing.of.cancer.cells	0.01706701	0.554592791
GO_AUTOPHAGIC CELL DEATH	Step1..release.of.cancer.cell.antigens	0.05929896	0.039907959
GO_AUTOPHAGIC CELL DEATH	Step2..cancer.antigen.presentation	0.085293092	0.003094571
GO_AUTOPHAGIC CELL DEATH	Step3..priming.and.activation	0.039404388	0.172352168
GO_AUTOPHAGIC CELL DEATH	T.cell.recruiting	-0.094464805	0.001046778
GO_AUTOPHAGIC CELL DEATH	CD4.T.cell.recruiting	-0.14175258	8.13E-07
GO_AUTOPHAGIC CELL DEATH	CD8.T.cell.recruiting	-0.121637997	2.37E-05
GO_AUTOPHAGIC CELL DEATH	Th1.cell.recruiting	-0.025191344	0.383073848
GO_AUTOPHAGIC CELL DEATH	Dendritic.cell.recruiting	-0.083109363	0.003949336
GO_AUTOPHAGIC CELL DEATH	Th22.cell.recruiting	-0.001576302	0.956480569
GO_AUTOPHAGIC CELL DEATH	Macrophage.recruiting	-0.19731843	5.24E-12
GO_AUTOPHAGIC CELL DEATH	Monocyte.recruiting	-0.253314814	4.86E-19
GO_AUTOPHAGIC CELL DEATH	Neutrophil.recruiting	-0.278812123	6.98E-23
GO_AUTOPHAGIC CELL DEATH	NK.cell.recruiting	-0.082492168	0.00422703
GO_AUTOPHAGIC CELL DEATH	Eosinophil.recruiting	-0.184157647	1.27E-10
GO_AUTOPHAGIC CELL DEATH	Basophil.recruiting	-0.276362316	1.70E-22
GO_AUTOPHAGIC CELL DEATH	Th17.cell.recruiting	-0.035658399	0.216881239
GO_AUTOPHAGIC CELL DEATH	B.cell.recruiting	0.06348336	0.027808837
GO_AUTOPHAGIC CELL DEATH	Th2.cell.recruiting	-0.007607013	0.792277959
GO_AUTOPHAGIC CELL DEATH	Treg.cell.recruiting	-0.148703111	2.26E-07
GO_AUTOPHAGIC CELL DEATH	MDSC.recruiting	-0.279558979	5.31E-23
GO_AUTOPHAGIC CELL DEATH	Step5..infiltration.of.immune.cells.into.tumors	0.073613726	0.010713019

GO_AUTOPHAGIC CELL DEATH	Step6..recognition.of.cancer.cells.by.T.cells	-0.040046545	0.165459413
GO_AUTOPHAGIC CELL DEATH	Step7..killing.of.cancer.cells	-0.079889961	0.005602739

Appendice Table A5. AUC of top 20 putative biomarkers.

Name	AUC	AUC CI
MFAP3	0.939	0.918-0.953
USP37	0.935	0.917-0.954
STAG1	0.934	0.913-0.95
ROCK1	0.934	0.912-0.953
DENND4C	0.933	0.908-0.951
ZNF770	0.931	0.904-0.955
ZBED6	0.93	0.911-0.946
ASXL2	0.929	0.91-0.948
TSTD2	0.928	0.904-0.946
USP34	0.926	0.901-0.949
SHPRH	0.922	0.899-0.945
ZNF699	0.921	0.895-0.945
ERCC6L2	0.92	0.898-0.938
NDUFA13	0.919	0.901-0.934
ZNF106	0.918	0.896-0.939
APC	0.918	0.895-0.939
ZNF490	0.916	0.891-0.942
NDUFA11	0.915	0.892-0.935
PHC3	0.915	0.892-0.936
GADD45GIP1	0.914	0.891-0.925
